# Intraoperative Electron Radiation Therapy (IOERT) in the management of locally recurrent rectal cancer

**DOI:** 10.1186/1471-2407-12-592

**Published:** 2012-12-11

**Authors:** Falk Roeder, Joerg-Michael Goetz, Gregor Habl, Marc Bischof, Robert Krempien, Markus W Buechler, Frank W Hensley, Peter E Huber, Juergen Weitz, Juergen Debus

**Affiliations:** 1 Clinical Cooperation Unit Radiation Oncology, German Cancer Research Center (DKFZ), Im Neuenheimer Feld 280, Heidelberg, 69120, Germany; 2 Department of Radiation Oncology, University of Heidelberg, Heidelberg, Germany; 3 Department of Radiation Oncology, Helios Clinic Berlin, Berlin, Germany; 4 Department of Surgery, University of Heidelberg, Heidelberg, Germany

**Keywords:** Recurrent, Rectal cancer, IOERT

## Abstract

**Background:**

To evaluate disease control, overall survival and prognostic factors in patients with locally recurrent rectal cancer after IOERT-containing multimodal therapy.

**Methods:**

Between 1991 and 2006, 97 patients with locally recurrent rectal cancer have been treated with surgery and IOERT. IOERT was preceded or followed by external beam radiation therapy (EBRT) in 54 previously untreated patients (median dose 41.4 Gy) usually combined with 5-Fluouracil-based chemotherapy (89%). IOERT was delivered via cylindric cones with doses of 10–20 Gy. Adjuvant CHT was given only in a minority of patients (34%). Median follow-up was 51 months.

**Results:**

Margin status was R0 in 37%, R1 in 33% and R2 in 30% of the patients. Neoadjuvant EBRT resulted in significantly increased rates of free margins (52% vs. 24%). Median overall survival was 39 months. Estimated 5-year rates for central control (inside the IOERT area), local control (inside the pelvis), distant control and overall survival were 54%, 41%, 40% and 30%. Resection margin was the strongest prognostic factor for overall survival (3-year OS of 80% (R0), 37% (R1), 35% (R2)) and LC (3-year LC 82% (R0), 41% (R1), 18% (R2)) in the multivariate model. OS was further significantly affected by clinical stage at first diagnosis and achievement of local control after treatment in the univariate model. Distant failures were found in 46 patients, predominantly in the lung. 90-day postoperative mortality was 3.1%.

**Conclusion:**

Long term OS and LC can be achieved in a substantial proportion of patients with recurrent rectal cancer using a multimodality IOERT-containing approach, especially in case of clear margins. LC and OS remain limited in patients with incomplete resection. Preoperative re-irradiation and adjuvant chemotherapy may be considered to improve outcome.

## Background

Despite major improvements in the treatment of primary rectal cancer, namely the introduction of neoadjuvant (chemo)-radiation and total mesorectal excision, locoregional recurrences still develop in about 5-15% of cases [1,2]. About 50% of these patients suffer from locally confined disease without distant spread [3] accompanied by high morbidity [4] and therefore represent candidates for a curative intent local treatment approach including surgical resection.

However, complete resections are difficult to achieve, because tumor growth is not confined to the initial anatomical compartments due to previous surgery [5]. The addition of external beam radiotherapy (EBRT) is also often limited, because many patients have already been exposed to radiotherapy during primary treatment and therefore the tolerance of the surrounding structures restricts dose prescription.

In this situation, IOERT as an adjunct to surgery could theoretically offer some advantages. First, the tumor bed representing the target volume, can be easily defined and restricted in size as no margins accounting for interfraction movements have to be added. Second, dose limiting structures like small bowel can be removed from the radiation field and protected from radiation side effects [6].

For these reasons, curative intent therapy for patients with recurrent rectal cancer included IOERT at our institution since 1991. In patients without prior irradiation IOERT was preceeded or followed by EBRT, otherwise IOERT was used as sole radiation treatment. The aim of the current analysis was to evaluate the clinical results in terms of disease control, overall survival and toxicity as well as prognostic factors in a retrospective manner.

## Methods

Between 1991 and 2006 a total of 113 patients suffering from locally recurrent rectal have been treated by surgery and IOERT at our institution. All patients gave written informed consent. 16 patients were excluded from analysis due to irresectable distant spread at the time of surgery or missing follow-up data. Patients with a history of prior metastasectomy or complete surgical removal of distant metastases during present surgery were included into the analysis. Patients charts and reports were reviewed to obtain patient and treatment characteristics. Regular follow-up examinations took place at our institution or at the referring centers. In case of missing follow-up, data was completed by calling the patient or the treating physician. The median follow-up for the entire cohort was 33 months (1–187 months) and 51 months in surviving patients.

The median time from first diagnosis to current treatment was 30 months (5–181 months). 83 patients were treated for their first local recurrence, 14 had multiple ones. 43 patients (44%) have had prior radiotherapy to the pelvis. For detailed characteristics of patients and treatment see Table 1. In general, local recurrence was discovered by routine follow-up or development of symptoms. It was confirmed histologically in the majority of patients before surgery, however in some cases diagnosis was based upon progressive growth on repeated CT- or MRI scans. Patients were scheduled for this treatment approach, if the risk for close or positive margins seemed high according to the surgeons assessment of preoperative imaging or after multidisciplinary discussion, especially if pelvic side wall or sacral involvement was present, whereas patients with limited, mainly intraluminal recurrences confined to the anastomotic region were usually treated with surgery alone. In all but two patients a radical resection was intended. Different surgical procedures were used at the discretion of the treating surgeon. In general, a trend to more extensive surgery including pelvic exenteration and partial resection of sacral bone was seen over time. In patients without a history of prior irradiation, surgery and IOERT were proceeded or followed by EBRT with a median dose of 41.4 Gy (range 15 to 54 Gy), usually in combination with simultaneous 5-FU based chemotherapy (89%). Assuming, that the biological effect of the large single dose used in IOERT is considered to be equivalent to 1.5-2.5 times the same total dose of fractionated RT [7], the EBRT dose of 41.4 Gy was chosen at the beginning of our IOERT program with the idea to reach dose escalation in the high risk area by the combination approach, while reducing late effects resulting from EBRT (i.e. small bowel obstruction) and IOERT (i.e. neuropathy) by combining moderate doses of each treatment as described in a previous publication by Eble et al. [8]. This concept emerged over time and since 2003, all unirradiated patients with recurrent rectal cancer were treated with EBRT doses of 45 to 54 Gy. Patients who received EBRT at our institution were usually treated in prone position on a belly board with a three-field technique, using three-dimensional conformal treatment planning routinely since 1995. The target volume included the entire tumor region, the perirectal, presacral and internal iliac node regions. Adjuvant chemotherapy was given only in a minority of patients (34%) at the discretion of the treating medical oncologist.

**Table 1:** Patient and treatment characteristics

**Patient characteristics**	**n**	**%**		**n**	**%**
Age at FD (yrs)			Prior RT		
Median	56		yes	43	44
Min	30		no	54	56
Max	74		Median dose (Gy)	50	
				
Age at IORT (yrs)			Distant metastasis prior to IORT		
Median	60		History of resected metastasis	13	13
Min	31		Resection during present surgery	5	5
max	78		both	2	2
			none	77	79
Time FD to IORT (mo)					
Median	30		Type of present surgery		
Min	5		AR	21	22
Max	181		APR	38	39
			Pelvic exenteration/bone resection	32	33
Gender			other	6	6
Male	59	61			
Female	38	39	Resection Status (present surgery)		
			R0	36	37
No. of recurrence			R1	32	33
First	83	86	R2	29	30
Multiple	14	14			
			EBRT in relation to present surgery		
T stage at FD			Neoadjuvant RT	6	6
T1	6	6	Neoadjuvant RCHT	40	41
T2	28	29	Adjuvant RT	0	0
T3	57	59	Adjuvant RCHT	8	8
T4	5	5	none	43	44
unknown	2	2			
			Adjuvant CHT		
N stage at FD			Yes	33	34
N0	54	56	No	64	66
N+	41	42			
unknown	2	2	IORT dose (Gy)		
			<10	1	1
M stage at FD			10	32	33
M0	92	95	12	18	19
M1 (all resected)	5	5	15	35	36
			18	8	8
UICC stage at FD			20	3	3
I	28	29			
II	25	26	IORT electron energy (MeV)		
III	37	38	Median	8	
IV	5	5	Min	6	
Unknown	2	2	Max	18	
Type of primary surgery			IORT cone size (cm)		
AR	65	67	Median	6.5	
APR	27	28	Min	5	
Local excision	5	5	Max	10	

n: number of patients, %: percentage, FD: first diagnosis, yrs: years, Min: minimum, Max: maximum, IORT: intraoperative radiation therapy, mo: Months, No.: number, UICC: Union internationale contre le cancer, RT: radiotherapy, AR: anterior resection, APR: abdominoperineal resection, R0: complete resection without microscopic residual disease, R1: microscopic residual disease, R2: gross residual disease, EBRT: external beam radiation therapy, RCHT: radiochemotherapy, CHT: chemotherapy, Gy: Gray, MeV: mega electron volts, cm: centimeter.

The technique of IOERT used at our institution has been described in detail earlier [6]. Briefly, IOERT was performed in a dedicated operation theatre with an integrated linear accelerator capable of delivering 6–18 MeV electrons. The target volume was defined in correspondence with the surgeon and included the high risk area for positive margins or visible residual tumor with a safety margin of 1 cm. The appropriate applicator was the placed manually and attached to the surgical table. Uninvolved radiosensitive tissues were displaced or protected by lead shields. The moveable table was located beneath the accelerator and properly aligned by a laser air-docking system in a focus-surface distance of 100 cm. In patients with additional EBRT, doses of 10–15 Gy were used intraoperatively, whereas patients with a history of prior irradiation received 15–20 Gy. Dose prescription was based on surgeons assessment of margin status including increasing but not routine use of intraoperative pathologic assessment of frozen sections during the overall study period. In general, higher IOERT doses were applied in cases suspicious of positive margins or residual disease according to the surgeons assessment. The dose was prescribed to the 90%-isodose. Central control (CC), local control (LC), distant control (DC), and overall survival (OS) were calculated from the date of surgery using the Kaplan-Meier method. CC and LC were defined as absence of tumor regrowth or progression inside the IOERT area or the pelvis, respectively. In patients without further assessment of LC e.g. after development of distant spread, the date of the last information about the local status was used for calculation. Differences in subgroups were tested for statistical significance by the log-rank test. Multivariate analysis was performed using the Cox regression method. Relations between distinct parameters were tested for significance by the Chi-square test. Differences were considered statistically significant for a p-value of ≤ 0.05.

The study is in compliance with the Declaration of Helsinki (Sixth Revision, 2008). Furthermore the study was approved by the Independent Ethics Committee of the Medical Faculty Heidelberg (Ref. Nr.: S-164/2012).

## Results

### Surgery

Complete resection, defined as microscopic negative resection margins in the final histopathological assessment, was achieved in 36 patients (37%), whereas 32 patients (33%) suffered from microscopic and 29 patients (30%) from macroscopic residual disease. The rate of complete resections was significantly linked to the use of neoadjuvant EBRT (52% vs. 24%, p=0.007). In contrast, the extent of the surgical procedure (pelvic exenteration and/or bony resection vs. anterior/abdominoperineal resection) had no statistically significant impact on resection margin.

### Central and local control

**Figure 1:**
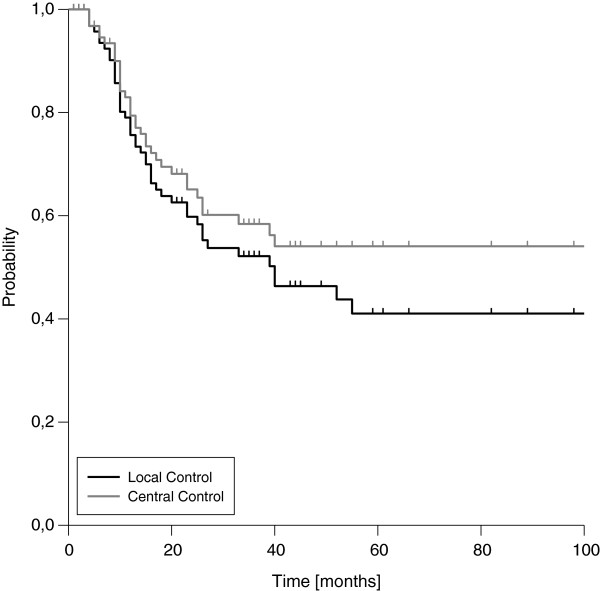
Central and Local Control (entire cohort)

**Figure 2:**
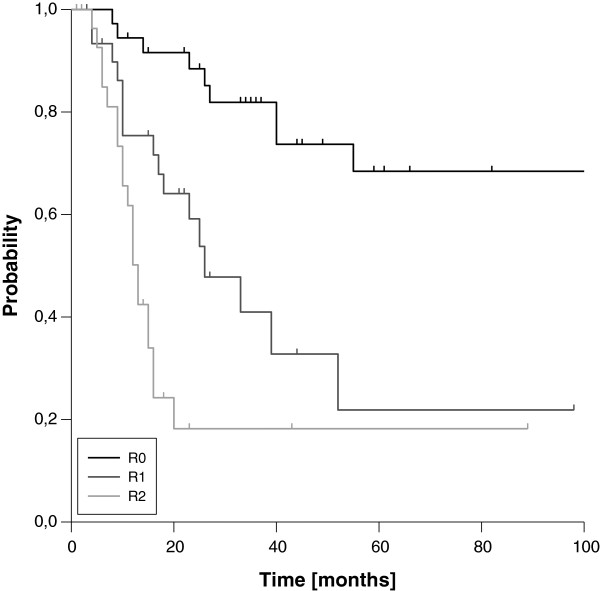
Local Control according to resection margin (R0 vs. R1 vs. R2)

The three- and five-year estimated LC rates for the entire cohort were 52% and 41%, respectively (Figure 1). The corresponding figures for CC were 58% and 54%, respectively. LC and CC rates were significantly correlated with resection margins. Whereas patients with complete resection (R0) showed three- and five-year LC rates of 82% and 68%, the three-year rates dropped to 41% and 18% in case of microscopic or macroscopic residual disease (Figure 2, Table 2), respectively. Of note, we found a significant difference in LC comparing patients with microscopic and macroscopic residual disease in univariate analysis (p=0.013). The influence of resection margins on LC remained statistically significant after correction for the use of additional EBRT (Table 3). Further on, female gender, negative nodal status and low clinical stage at first diagnosis were significantly correlated with improved LC in univariate analysis. Trends to improved LC were seen for the use of neoadjuvant EBRT in general and for IOERT doses ≥15 Gy in patients with microscopic residual disease (Table 2). In the multivariate model, the strong impact of resection margin on LC could be confirmed (Table 4), but none of the other factors remained statistically significant. Interestingly, the time interval between first diagnosis and recurrence reached statistical significance in the multivariate model, although we did not found a significant impact in univariate analysis. A trend to improved LC was observed for the use of additional EBRT in the multivariate model.

**Table 2:** Univariate analysis of prognostic factors

**Univariate analysis**	**n**	**%**	**3-y-LC**	**p-value**	**3-y-DC**	**p-value**	**3-y-OS**	**p-value**
all patients	97	100	52%		48%		52%	
age								
< 60 yrs	47	48	49%	0.329	50%	0.522	48%	0.619
≥ 60 yrs	50	52	55%		47%		55%	
gender								
male	59	61	44%	**0.023**	52%	0.779	53%	0.701
female	38	39	65%		42%		54%	
time FD to rec								
< 30 mo	48	49	49%	0.308	46%	0.389	43%	0.398
≥ 30 mo	49	51	55%		50%		61%	
T stage (FD)*								
T1/2	34	35	64%	0.13	61%	0.056	70%	**0.045**
T3/4	62	64	43%		40%		43%	
N stage (FD)*								
N0	54	56	61%	**0.008**	53%	**0.047**	60%	0.06
N+	41	42	39%		42%		37%	
UICC stage (FD)*								
I/II	53	55	60%	**0.015**	55%	**0.03**	62%	**0.046**
III/IV	42	43	41%		41%		41%	
resection margin								
R0	36	37	82%	**< 0.001**	55%	**0.047**	80%	**< 0.001**
R1	32	33	41%		42%		37%	
R2	29	30	18%		48%		35%	
EBRT								
yes	54	56	59%	0.062	49%	0.928	60%	0.18
no	43	44	41%		50%		43%	
EBRT								
neoadj RT	46	47	61%	0.067	51%	0.797	59%	0.284
no RT	43	44	41%		50%		43%	
adj. CHT								
yes	33	34	50%	0.817	54%	0.945	63%	0.745
no	64	66	54%		47%		45%	
resection of met								
yes	20	21	65%	0.358	35%	0.155	47%	0.484
no	77	79	49%		52%		54%	
local control achieved								
yes	52	54	not amendable		53%	0.2	55%	**0.03**
no	45	46			43%		52%	

n: number of patients, %: percentage, 3-y-LC: three-year local control, 3-y-DC: three-year distant control, 3-y-OS: three-year overall survival, yrs: years, FD: first diagnosis, rec: local recurrence, mo: months, *: based on 95 patients with known stage at first diagnosis, UICC: Union internationale contre le cancer, R0: complete resection without microscopic residual disease, R1: microscopic residual disease, R2: gross residual disease, EBRT: external beam radiation therapy, RT: radiotherapy, adj.: adjuvant, CHT: chemotherapy, met.: distant metastasis.

**Table 3:** Prognostic value of resection margin for LC and OS with or without EBRT (univariate analysis)

	**EBRT + IOERT**	**IOERT alone**	**EBRT + IOERT**	**IOERT alone**
	**3-y-LC**	**3-y-LC**	**3-y-OS**	**3-y-OS**
**R0**	83%	79%	72%	100%
**R1**	52%	29%	43%	31%
**R2**	13%	21%	53%	17%
**p-value**	<0,001	0,028	0,011	<0,001

LC: local control, OS: overall survival, EBRT: external beam radiation therapy, IOERT: intraoperative electron radiation therapy, 3-y-LC: three-year local control, 3-y-OS: three-year overall survival, R0: complete resection without microscopic residual disease, R1: microscopic residual disease, R2: gross residual disease, %: percentage.

**Table 4:** Significant prognostic factors in multivariate analysis

**prognostic factor**	**Local control**		**Overall Survival**	
	**hazard ratio**	**p-value**	**hazard ratio**	**p-value**
**time FD to rec**				
< 30 months	1		1	
≥ 30 months	0.95 (0.92-0.98)	**0.04**	0.98 (0.96-1.01)	0.221
**resection margin**				
R0	1		1	
R1	7.12 (2.29-22.12)	**0.001**	10.12 (3.76-27.27)	**< 0.001**
R2	23.25 (7.22-74.79)	**< 0.001**	13.04 (4.44-38.38)	**< 0.001**

FD: first diagnosis, rec: local recurrence, R0: complete resection without microscopic residual disease, R1: microscopic residual disease, R2: gross residual disease values in parentheses: 95% confidence intervals.

### Overall survival

**Figure 3:**
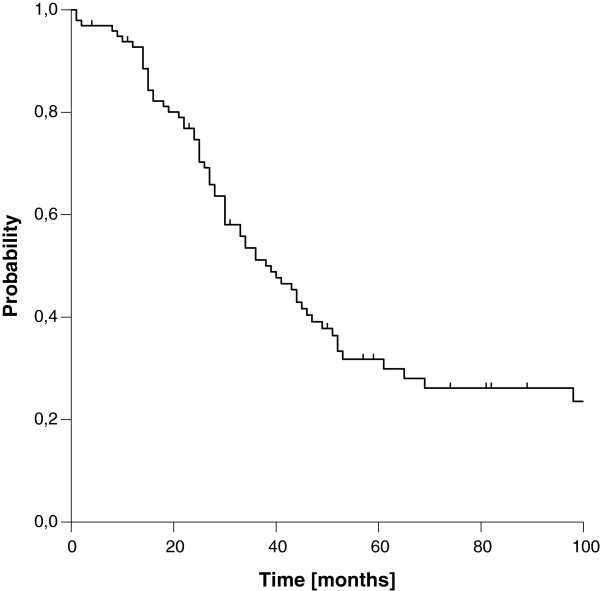
Overall Survival (entire cohort)

**Figure 4:**
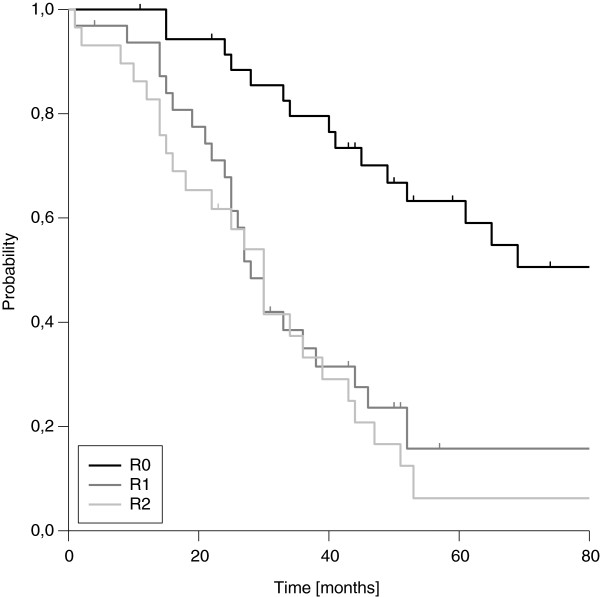
Overall Survival according to resection margin (R0 vs. R1 vs. R2)

**Figure 5:**
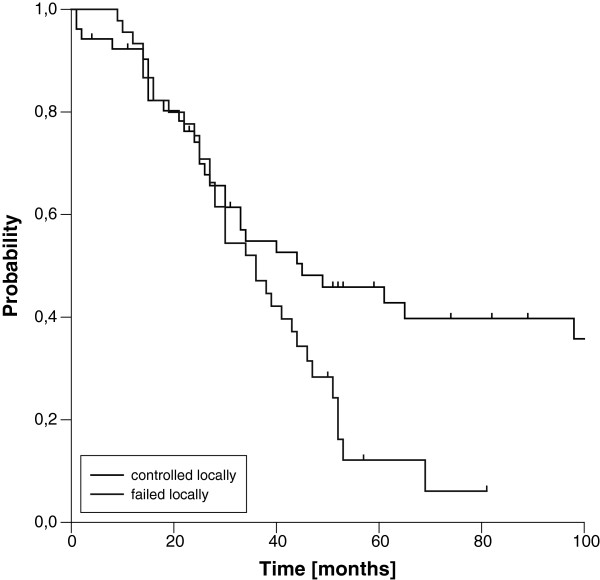
Overall survival according to local control after current treatment (locally controlled vs. local failure)

We found a median estimated OS of 39 months, transferring into three- and five-year estimated OS rates of 52% and 30%, respectively (Figure 3). OS was strongly influenced by resection margins. Whereas patients with microscopic complete resection showed very favourable three- and five-year OS rates of 80% and 63%, the three- and five-year rates dropped to 36% and 11% in case of incomplete resection, respectively (Figure 4). In contrast to LC, we did not observe a difference in OS according to the extent of residual disease. Again, the influence of margin status remained statistically significant after correction for the use of additional EBRT (Table 3). OS was further significantly affected by T stage and clinical stage at first diagnosis in univariate analysis. A trend to improved OS was seen in node-negative patients at first diagnosis (Table 2). In the multivariate model, resection margin remained the only factor with significant impact on OS (Table 4). If patients with an achievement of local control were compared to patients with a re-recurrence after IOERT, a significant benefit in terms of OS was observed, which manifested after 3 years (Figure 5).

### Distant control

**Figure 6:**
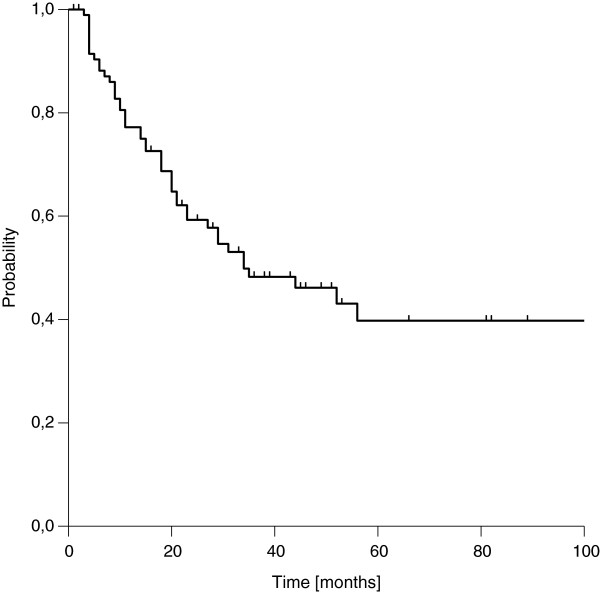
Distant Control (entire cohort)

The estimated three-and five-year DC rates for the entire cohort were 48% and 40%, respectively (Figure 6). Resection margin, nodal status at first diagnosis and clinical stage at first diagnosis were significantly associated with DC in univariate analysis (Table 2). A trend was seen for T stage at first diagnosis. In the multivariate model only resection margin was significantly associated with DC. Trends could also be found for the time interval between first diagnosis (p=0.074) and recurrence and for a history of metastasectomy (p=0.1). Of note, the first occurrence of distant metastasis after the present treatment was most frequently located in the lung (37%) (Table 5).

**Table 5:** Distribution of distant metastases

**Distant metastasis (first site of distant failure)**	**n**	%*
lung	17	37
liver	8	17
peritoneum	6	13
lymph node°	5	11
bone	4	9
brain	2	4
multiple sites	4	9

n: number of patients, %: percentage, *: based on 46 patients with distant failure after current treatment, °: non-regional.

### Toxicity

The 90-day perioperative mortality rate was 3.1%. A total of 80 complications occurred in 57 patients (59%). The most common complications were postoperative wound healing disturbances and abscess/fistula formation, which have been observed in 20% and 16% of the patients, respectively. These complications caused also the majority (77%) of the 22 surgical re-interventions (including incisions or drainage procedures). Another common side effect was urinary retention, which was found in 12% of the patients, but resolved after a short time period in the majority of cases. Peripheral neuropathy including severe chronic pain was observed in 8% of the patients (Table 6). Neuropathy was found in 11% of the patients receiving IOERT doses of ≥15 Gy compared to 6% in patients with <15 Gy, but this difference was not statistically significant.

**Table 6:** Complications and toxicity

**Complications and Toxicity‘**	**n**	**%**
wound healing disturbance	19	20
abscess or fistula	16	16
hemorrhage	6	6
ureteral stenosis	3	3
anal stenosis	2	2
ileus	3	3
neuropathy (incl. chronic pain)	8	8
urinary retention (transient)	9	9
urinary retention (persistent)	3	3
delayed gastrointestinal passage	3	3
perineal hernia	1	1
arterial catheter abcess	1	1
severe sphincter insufficiency	1	1
urinary leakage	1	1
compartment syndrome	1	1
pleural effusion	1	1
venous thrombosis	1	1
pulmonary embolism	1	1

N: number of patients, %: percentage, ‘: multiple events in some patients.

## Discussion

Improvements in multimodality treatment of primary rectal cancer resulted in a reduction in local recurrences, however considering the widely exhausted tolerances of the surrounding tissues by previous surgery and radiotherapy, their treatment has become even more challenging and sometimes has led to a nihilistic approach considering this situation as palliative only. In contrast, this report describes a large single-center experience using a multimodality IOERT-containing regimen, which shows that long term LC and OS is achievable in a substantial proportion of patients, although conclusions should be drawn with caution regarding the known limitations of retrospective analyses in general and especially in terms of possible selection biases.

Conservative treatment approaches including palliative EBRT usually result in a median survival <12 months and 5-year OS rates <5% [3,9]. With surgery alone, 5-year OS rates in the range of 20-35% [10-13] have been observed if free margins were achieved, but long term survival was almost absent in patients with residual disease [9,10,14]. Because the rate of complete resections in patients undergoing surgery is only in the range of 30-45% [3], additional irradiation has been investigated. Response to neoadjuvant EBRT has been shown to result in increased complete resection rates and decreased local failure rates in primary rectal cancer [1,15]. Similar effects have been also described for recurrent rectal cancer [5,14]. However, the dose of conventionally fractionated EBRT required for control of residual disease is estimated to be ≥60 Gy and therefore exceeds small-bowel tolerance already in previously not irradiated patients [16,17]. Further on, an increasing number of patients suffering from recurrent rectal cancer had already been previously irradiated and concerns about toxicity prevented many groups from the use of external beam re-irradiation [4]. Therefore IOERT has been investigated because of the opportunity to remove critical organs at risk from the target volume [6]. In cases without prior irradiation, the combination with EBRT also allows safe dose escalation to overcome the dose limitations of EBRT alone as shown in advanced primary rectal cancer [18].

In our series, patients without prior irradiation received EBRT combined with 10–15 Gy IOERT. Patients with prior irradiation have been treated with IOERT alone. No re-irradiation was performed. We observed a median survival of 39 months with a 5-year OS rate of 30% and a 5-year LC rate of 41%. In patients with microscopic complete resection, very favourable 5-year OS and LC rates of 63% and 68% were found. Incomplete resection (microscopic or gross) was clearly associated with a worse outcome (5-year OS and LC rates of 11% and 19% for the combined group, respectively). Our results compare favourably with the series using surgery alone, especially in patients with complete resection and are in line with the findings of other groups investigating IOERT-containing approaches. Haddock et al. [4] described the same 5-year OS of 30% and an extraordinary 5-year LC rate of 72% in the largest series ever published. Dresen et al. [5] found a 5-year OS of 31.5% and a 5-year LC rate of 50% in a large cohort from the Netherlands. Lindel et al. [19] observed 3-year OS and LC rates of 27% and 31% in a series from MGH and Alektiar et al. [20] described 5-year OS and LC rates of 23% and 39% in a MSKCC series using HDR-IORT.

However, resection margin remained the strongest prognostic factor for LC and OS in our and other major series (Table 7). Obviously, IOERT does not thoroughly compensate for an incomplete resection, with 5-year OS rates of 36-63% and LC rates of 43-72% after microscopic complete resection compared to 5-year OS rates of roughly 10-30% after incomplete resection. Therefore efforts should be made to increase the rate of R0 resections. We observed a significantly increased rate of free margin resections in patients who were able to receive neoadjuvant EBRT. Dresen et al. [5] described a similar association and Saito et al. [12] found a statistically increased LC and even OS rate after neoadjuvant EBRT compared to surgery alone. Undoubtly, the use of EBRT is feasible in previously untreated patients, but given the somewhat disappointing results in previously irradiated patients with incomplete resection using IOERT alone in our and other series [5,21,22], renewed interest should be paid to the possibility of re-irradiation. In contrast to major concerns about severe side effects, re-irradiation with moderate doses of 30–40 Gy has been associated with acceptable toxicity [23-26], if restricted target volumes were used and the interval to previous irradiation was >6 months. In these four reports focussing on reirradiation, a total of 270 previously irradiated patients have been included and received conventional or hyperfractionated EBRT with median doses ranging 23.4 to 40.8 Gy, mainly combined with concurrent chemotherapy. Resection rates varied from 33% [25] to 75% [23]. Two groups also used IORT with median doses of 10–15 Gy in 50% of the resected patients [23,26]. Median follow up times ranged from 24 months to 82 months [23-26]. Severe (grade 3/4) acute toxicity rates ranged from 4%-28% [23-26] and severe late side effects were observed in 11%-26% [24-26]. The observed 5-year LC and 5-year OS rates were 33-39% [24,26] and 19%-40% [23-26] for the entire groups, respectively. In the subgroups of patients with resection of recurrent disease (irrespective of margin status), the 5-year OS rates seemed improved with 22%-54% [23,25,26], compared to unresected patients with 5-year OS rates of 0%-22% [23-26]. The best results have been observed in patients with R0-resection after re-irradiation with 5-year LC and OS rates of 69%-70% and 67%-72% in the two series from Italy [23,24], in which R0 resection was also strongly associated with LC and OS in multivariate analysis.

**Table 7:** Results after IORT in R0 and R+ patients (only series >50 patients included)

**Study**	**R0**			**R+**		
	**n**	**5-year OS**	**5-year LC**	**n**	**5-year OS**	**5-year LC**
Haddock et al. (2011) [4]	226	46	72	380	27/16 (R1/2)	68
Dresen et al. (2008) [5]	84	48	69	63	12	29
Lindel et al. (2001) [19]	25	40	56	24	14	17
Alektiar et al. (2000) [20]	53	36	43	21	7 (R1)	16 (R1)
Wiig et al. (2002) [27]	18	60	70	41	20/0 (R1/2)	50/0 (R1/2)
Current study	33	63	68	64	11	19

IORT: intraoperative radiation therapy, n: number of patients, R0: complete resection without microscopic residual disease, R+: microscopic or gross residual disease, 5-year OS: 5-year overall survival [percentage], 5-year LC: 5-year local control [percentage], R1: microscopic residual disease, R2: gross residual disease.

Although considering probable variations in patient selection given the wide range in the percentage of resected patients and overall outcome in those reports, the possible benefit of neoadjuvant re-irradiation was consistently mainly confined to patients achieving resectability (especially with free margins), whereas outcome of patients with gross residual disease or no resection at all remained dismal. Therefore neoadjuvant re-irradiation seem to improve outcome mainly by increasing the rate and completeness of following surgery, which is supported by the strong and significant association between resection or margin status and overall survival in uni- and/or multivariate analysis in all of series [23-26]. The value of neoadjuvant re-irradiation in terms of increased resectability was also confirmed by Dresen et al. [5], who described significantly increased rates of complete resections after neoadjuvant re-irradiation, which transferred into improved LC and OS in combination with IOERT compared to IOERT alone.

Assuming the dose, that can be safely delivered in previously irradiated patients with EBRT, is probably limited to 30–40 Gy mainly due to bowel toxicity (which was the main side effect in the large series published by Mohiuddin et al. [25]), and the dose that can be safely applied thereafter via IOERT (with the opportunity to exclude bowel from the irradiation field) is probably limited to about 15 Gy mainly due to neuropathy [28], the combination of both approaches might be the best idea, as advocated by investigators from Mayo [4] and Eindhoven [5]. In summary, given the morbidity of uncontrolled locoregional disease and the finding from our series, that the achievement of LC in general is associated with significantly improved overall survival, the consideration of external beam re-irradiation followed by surgery and IOERT seems justified.

Unfortunately, patients with recurrent rectal cancer are also at high risk for distant failure. We observed a 5-year-DC rate of 40% in our patients. Similar results have been shown in the series from Mayo (5-year-DC rate 47%) [4] and the Netherlands (5-year-DC 50%) [5], in which adjuvant systemic treatment was also uncommon. In contrast to primary rectal cancer patients, the lung was the most common site in our cohort, probably due to the changed venous drainage caused by the surgical intervention during primary treatment. Similar patterns of distant failures were described by other investigators [28]. This may indicate, that at least a substantial proportion of distant failures were caused through disseminating tumor cells from the recurrence rather than being linked to the primary disease. This association is also supported by the prognostic value of resection margin for distant control in many series including ours [4,5]. However, we also observed a statistically significant impact of TNM stage in primary situation on outcome. Similar results were found by other investigators [5,29]. This may indicate, that an unfavourable biology of the disease (expressed by a locally advanced stage in primary situation) could also have caused a worse outcome. We could not confirm the value of adjuvant chemotherapy in our series, however this result could have been biased because patients with adverse prognostic factors were more likely to receive it. Nevertheless, given the high rates of distant metastasis including the changes in their patterns, the introduction of adjuvant systemic therapy seems reasonable. This assumption is also supported by Hashiguchi et al. [30], who observed a significantly improved OS in patients treated with adjuvant chemotherapy after resection and IOERT.

## Conclusion

In summary, multimodality treatment including surgery, IOERT and EBRT resulted in encouraging LC and long term OS in a substantial proportion of locally recurrent rectal cancer patients, especially if free margins could be achieved. Neoadjuvant EBRT in previously untreated patients resulted in increased rates of complete resections, which remained the strongest prognostic factor for disease control and overall survival. Currently we use 45–50.4 Gy in combination with chemotherapy followed by surgery and IOERT with 10–15 Gy in patients without prior irradiation. Given the limited results in previously irradiated patients after incomplete resection with IOERT alone, additional re-irradiation should be considered in carefully selected patients, especially since achievement of local control seemed crucial for long term survival. Intensified adjuvant systemic treatment may be warranted given the high numbers of distant failures.

## Competing interest

The authors declare that they have no conflicts of interest.

## Authors’ contributions

FR planned the analysis, participated in data acquisition, data analysis, literature review, patient treatment, and drafted the manuscript. JMG performed main parts of data acquisition, data analysis, literature review and participated in manuscript draft. GH, MB, RK, MWB, FWH and PEH participated in data aquisition, data analysis, literature review and patient treatment. JD and JW participated in planning of the analysis, patient treatment and revised the manuscript critically. All authors read and approved the final manuscript.

## Pre-publication history

The pre-publication history for this paper can be accessed here:

http://www.biomedcentral.com/1471-2407/12/592/prepub
